# Prenatal hypoxia-induced epigenomic and transcriptomic reprogramming in rat fetal and adult offspring hearts

**DOI:** 10.1038/s41597-019-0253-9

**Published:** 2019-10-29

**Authors:** Xin Chen, Lubo Zhang, Charles Wang

**Affiliations:** 1 Center for Genomics, School of Medicine, Loma Linda University, Loma Linda, CA 92350 USA; 2 Department of Basic Sciences, School of Medicine, Loma Linda University, Loma Linda, CA 92350 USA; 3 Lawrence D. Longo, MD Center for Perinatal Biology, School of Medicine, Loma Linda University, Loma Linda, CA 92350 USA

**Keywords:** Rat, Heart development, DNA methylation, Transcriptomics, Sequencing

## Abstract

The molecular mechanism of antenatal hypoxia impacting on fetal heart development and elevated risk of heart disease of adult offspring is poorly understood. We present a dataset integrating DNA methylome and transcriptome analyses of antenatal hypoxia affecting rat fetal and adult offspring hearts to understand hypoxia-mediated epigenomic reprogramming of the heart development. We showed that antenatal hypoxia not only induced DNA methylomic and transcriptomic changes in the fetal hearts, but also had a delayed and lasting effect on the adult offspring hearts. Of interest, antenatal hypoxia induced opposite changes in DNA methylation patterns in fetal and adult hearts, with a hypermethylation in the fetus and a hypomethylation in the adult. An extensive preprocessing, quality assessment, and downstream data analyses were performed on the genomic dataset so that the research community may take advantage of the public resource. These dataset could be exploited as a comprehensive resource for understanding fetal hypoxia-mediated epigenetic reprogramming in the heart development and further developmental programming of heart vulnerability to disease later in life.

**Figshare doi**: 10.6084/m9.figshare.9948572

## Abstract (summary)

**Table:** 

Measurement(s)	transcription profiling assay • DNA methylation
Technology Type(s)	RNA sequencing • RRBS
Factor Type(s)	sex • developmental stage • treatment
Sample Characteristic - Organism	Rattus norvegicus

Machine-accessible metadata file describing the reported data: 10.6084/m9.figshare.9948572

## Background & Summary

Heart disease is the leading cause of death in the United States, with ischemic heart disease, a major cause of morbidity and mortality. Yet the molecular mechanisms remain largely elusive. In addition to other risk factors, large epidemiological and animal studies have shown a clear association of adverse intrauterine environment with increased risk of ischemic heart disease in adulthood^1–5^. Hypoxia is a common form of intrauterine stress, and the fetus may experience prolonged hypoxic stress under a variety of conditions, including pregnancy at high altitude, pregnancy with anemia, placental insufficiency, cord compression, preeclampsia, heart, lung and kidney disease, or with hemoglobinopathy. Previous studies have suggested a possible link between antenatal hypoxia and increased risk of cardiovascular disease in offspring^6–16^. Studies in rats have demonstrated that maternal hypoxia results in heightened cardiac vulnerability to ischemia and reperfusion injury in offspring^17–25^. DNA methylation is a key mechanism associated with human diseases, altered gene expression and phenotype. Emerging evidence suggests that DNA methylation may play a crucial role in gestational hypoxia-induced developmental reprogramming. Consistent with what we reported recently in animal model^26^, there were other studies also showing that a chronic hypoxia induced DNA methylation alteration and/or transcriptomic reprogramming in cardiomyocytes^27,28^. Our previous studies showed that hypermethylation of single gene (e.g., PKC) was involved in antenatal hypoxia-induced developmental plasticity^21,24^. However, the impact of fetal hypoxia on the alteration of global methylation pattern and transcriptomic changes in the heart development remain unclear.

Recently, we presented an integration analysis to utilize reduced-representation bisulfite sequencing (RRBS) DNA methylome analysis coupled with RNA-seq to test the hypothesis that antenatal hypoxia causes a global epigenomic reprogramming and a corresponding transcriptomic alteration in developmental programming of hypoxic/ischaemic-sensitive phenotype in the heart^26^. The study showed that antenatal hypoxia not only induced a global DNA methylomic and transcriptomic changes in the fetal hearts, but also had a delayed and lasting effect on the heart in adult offspring^26^. In summary, our DNA methylome analyses identified 2,828 (1,824 hyper- vs. 1,004 hypo-) differentially methylated regions (DMRs) in fetal hearts and 2,193 (647 hyper- vs. 1,546 hypo-) DMRs in adult male hearts between hypoxia exposure and control. The differential methylation analysis showed an inverse hyper-hypo proportion relationship between fetal and adult male hearts. Consistent with the differential methylation analysis, an opposite global DNA methylation pattern caused by antenatal hypoxia was observed between fetal and adult hearts when examining the transcript starting site regions (TSS ± 3 k) and CpG islands (CGIs) in all genes, which displayed a hypermethylation in the fetal hearts and a hypomethylation in the adult hearts. Our transcriptome analysis identified a total of 323 differentially expressed genes (DEGs) in fetal hearts and 112 DEGs in adult male hearts between prenatal hypoxia exposure and control. More up-regulated DEGs were identified in fetal (62.2%), compared to adult male rats (37.5%). Our pathway analysis based on the DEGs showed a significant difference between fetal and adult male rats. In fetal hearts, developmental/stress response related pathways were enriched, whereas in adult male rat hearts, immune/inflammatory response related pathways were enriched. Our integration analysis on DNA methylome and transcriptome data revealed a strong negative correlation between TSS CpG methylation and gene expression using the pooled data from both fetal and adult hearts. Furthermore, there was a strong negative correlation between DNA methylation and gene expression level when DMRs were located in promoter or exon regions of genes. We would like to note that our dataset including both DNA methylome and RNAs-seq transcriptome in a rat model involving prenatal hypoxia exposure is very unique and rare in the community which will have a great value and reusability. The dataset is in a high quality. More biological insights can be derived with further data mining performed by different investigators. Particularly, our dataset has special value to the those studying the maternal exposure induced epigenomic and transcriptomic reprogramming in the fetus.

## Methods

We have presented some these methods in our primary publication^26^. This section expanded our previous description to provide a comprehensive resource for reproducing both experimental and computational analysis.

### Experimental study design

**Fig. 1:**
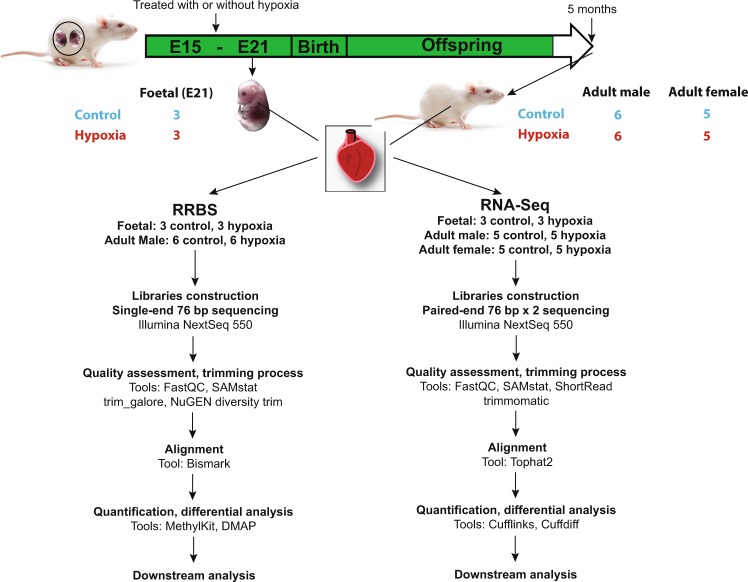
Flowchart of experimental design and data analysis pipeline.

The overall experimental study design was illustrated in Fig. 1. In total, we constructed 18 RRBS methylation libraries derived from 18 Sprague Dawley (SD) rat hearts and 26 RNA-seq libraries derived from 26 SD rat hearts in two development stages (fetus and adult). Pregnant rats were exposed to either normoxia (control group) or hypoxia (10.5% O2) for 6 days during gestational Days 15–21. The fetal hearts were collected at the Day 21 of gestation, and the adult offspring hearts were collected at 5 months old after birth^26^.

### DNA and RNA extractions

The methods for DNA and RNA extractions were described previously^26^. Briefly, for genomic DNA (gDNA) extraction, heart tissues were minced and digested with 20 μL proteinase K (20 mg/mL) in digestion buffer (10 mM Tris-Hcl, pH 8.0 containing 0.5 M EDTA, 1% SDS, 10 m NaCl, and 5 mM CaCl_2_) for 3 h at 55 °C. The tissue lysate was twice phenol/chloroform/isoamyl: (25/24/1) alcohol extracted and the aqueous layer was treated with RNase A + T1 for 1 h at 37 °C. The lysate was again phenol/chloroform/isoamyl alcohol extracted and the gDNA was precipitated with equal volume of isopropanol in presence of 0.3 M sodium acetate at −20 °C overnight. The gDNA was finally centrifuged, washed with 70% ethanol, air dried and reconstituted in 10 mM Tris-HCl, pH 8.0. And then, gDNA was denatured with 2 N NaOH at 42 °C for 15 min, treated with sodium bisulfite at 55 °C for 16 h, and purified by EZ DNA Methylation-Gold KitTM (Zymo Research, Tustin, CA, USA)^26^.

Total RNA was isolated from heart tissues using TRIzol RNA Isolation reagent (Life Technologies, Carlsbad, CA, USA). Briefly, each solid tissue was homogenized in 1 mL TRIzol and the lysate was then extracted with chloroform and centrifuged at 14,000 rpm for 15 min at 4 °C. Finally, total RNA was precipitated and washed thoroughly with 70% ethanol, air dried and reconstituted in nuclease free water. RNA quality was assessed using the 2200 TapeStation (Agilent Technologies, Wilmington, DE USA). Heart samples had an average RIN number of 9.5^26^.

### RRBS and RNA-seq library constructions

The methods for RRBS and RNA-seq library constructions were described previously^26^. Briefly, high-quality gDNA was used for generation of RRBS libraries at the Center for Genomics, Loma Linda University (Loma Linda, CA, USA) following standard protocols of the Ovation® Ultralow Methyl-Seq Library Systems (NuGEN Technologies, Inc. USA). Briefly, 100 ng gDNA was restriction digested at 37 °C for 1 hour using the methyl-insensitive restriction enzyme Mspl, which cuts the DNA at CCGG sites. The fragments were directly subject to end blunting and phosphorylation. A single nucleotide (A) was then added to the 3′ ends of the fragments in preparation for ligation to a methylated adapter with a single-base T overhang. The ligation products were final repaired in a thermal cycler under the program (60 °C – 10 min, 70 °C – 10 min, hold at 4 °C). The product of the final repair reaction can be input directly into the bisulfite conversion kit (QIAGEN EpiTect Fast DNA Bisulfite Kit) according to Qiagen’s protocol. Eluted the purified, bisulfite-converted DNA in 23 μL of EB and performed PCR-amplification to enrich for fragments with adapters on both ends following by Agencourt RNAClean XP Beads purification. The final libraries were quantified using Qubit 3.0 (Life Technologies, Carlsbad, CA, USA) and the average size was determined on an Agilent TapeStation 2200 (Agilent Technologies, Wilmington, DE, USA). The final library was diluted to 5 nM and further quantitated to ensure high accuracy quantification for consistent pooling of barcoded libraries before sequencing^26^.

RNA-seq libraries were constructed using the Ovation Universal RNAseq System (NuGEN, San Carlos, USA). All RNA samples (except non-template control) were spiked with 1:500 ERCC RNA spike-in control mix (Life Technologies). Before cDNA generation, samples were treated with a second round of DNase for more thorough removal of gDNA following NuGEN’s integrated DNase treatment protocol. Double-stranded cDNA was generated using ~100 ng of total RNA per sample. cDNA was sheared using the Covaris S220 sonication system. Each sample was sheared according to the manufacturer’s settings of 130 µL sample with a target (peak) of 200 bp. The settings were as follows: 10% duty factor and 200 cycles/burst at 7 °C for 180 seconds. End repair was then performed to generate blunt ends for adaptor ligation. Unique barcodes were used for each sample for multiplexing. Targeted rRNA-depletion was performed before final library construction. cDNA libraries were amplified using 15 cycles (Artik thermal cycler from Thermo Scientific) and purified using RNAClean XP beads (Agencourt, Brea, USA). The size distribution of the libraries was checked using 2200 TapeStation. The peak size for all samples was around 300 bp (including a 122 bp adaptor). All libraries were quantified using the Qubit 3.0 Fluorometer (Life Technologies) and stored at −20 °C in non-sticky Eppendorf tubes (Life Technologies, Carlsbad, USA). RNA-seq libraries were sequenced on Illumina NextSeq550 with 76 bpx2, PE, approximately 25 M reads/each, at the Center for Genomics, Loma Linda University^26^.

### Sequencing, processing, and alignment of RRBS and RNA-seq libraries

RRBS libraries were sequenced using Illumina Nextseq550 platform. The libraries were single-end (SE) and read length was 76 bp. Base-calling was performed by Illumina RealTime Analyzer (RTA) software. Binary base call files (bcl) were demultiplexed by bcl2fastq v2.17.1.14 to generate raw fastq data per sample. Quality of the raw fastq data for each sample was assessed using the FastQC v0.11.4^29^. Based on the quality, adaptor sequences were removed from the reads using trim_galore v0.3.7^30^ with option ‘–rrbs’ disabled due to special design of NuGEN Ovation RRBS system. After adaptor trimming, NuGEN’s diversity trimming was performed to remove diversity adaptors. The trimmed reads were aligned to rat genome NCBI Rnor6.0, downloaded from Illumina iGenome, by using Bismark v0.16.3 with all default parameter settings. The methylation call files including the location of each CpG sites and the methylation percentage (beta value) were generated using bismark_methylation_extractor. SAMstat v 1.5.1^31^ was used to assess the quality of aligned reads per sample. The detailed statistics summary of RRBS processing and alignment can be referred to Supplementary Table 1.

RNA-seq libraries were sequenced on Illumina Nextseq550 with paired-end, 76 bpx2 run. FastQC and ShortRead^32^ (R/Bioconductor package) were used to assess the quality of the raw fastq data per sample. Based on the quality, hard trimming and adaptor trimming were performed by Trimmomatic v0.35^33^. The trimmed reads were aligned to the rat reference genome NCBI Rnor6.0 with TopHat v2.1.1^34^ with default parameter settings. The aligned bam files were then processed using Cufflinks v 2.2.1^35^ for gene quantification. Reads that were unable to align to the rat genome were converted to fastq format using SamToFastq function in Picard v1.114, and further mapped and quantified to ERCC transcripts by TopHat and Cufflinks, respectively. The detailed statistics summary of RNA-seq processing and alignment can be referred to Supplementary Table 2.

### Differential analysis of RRBS and RNA-seq data

**Fig. 2:**
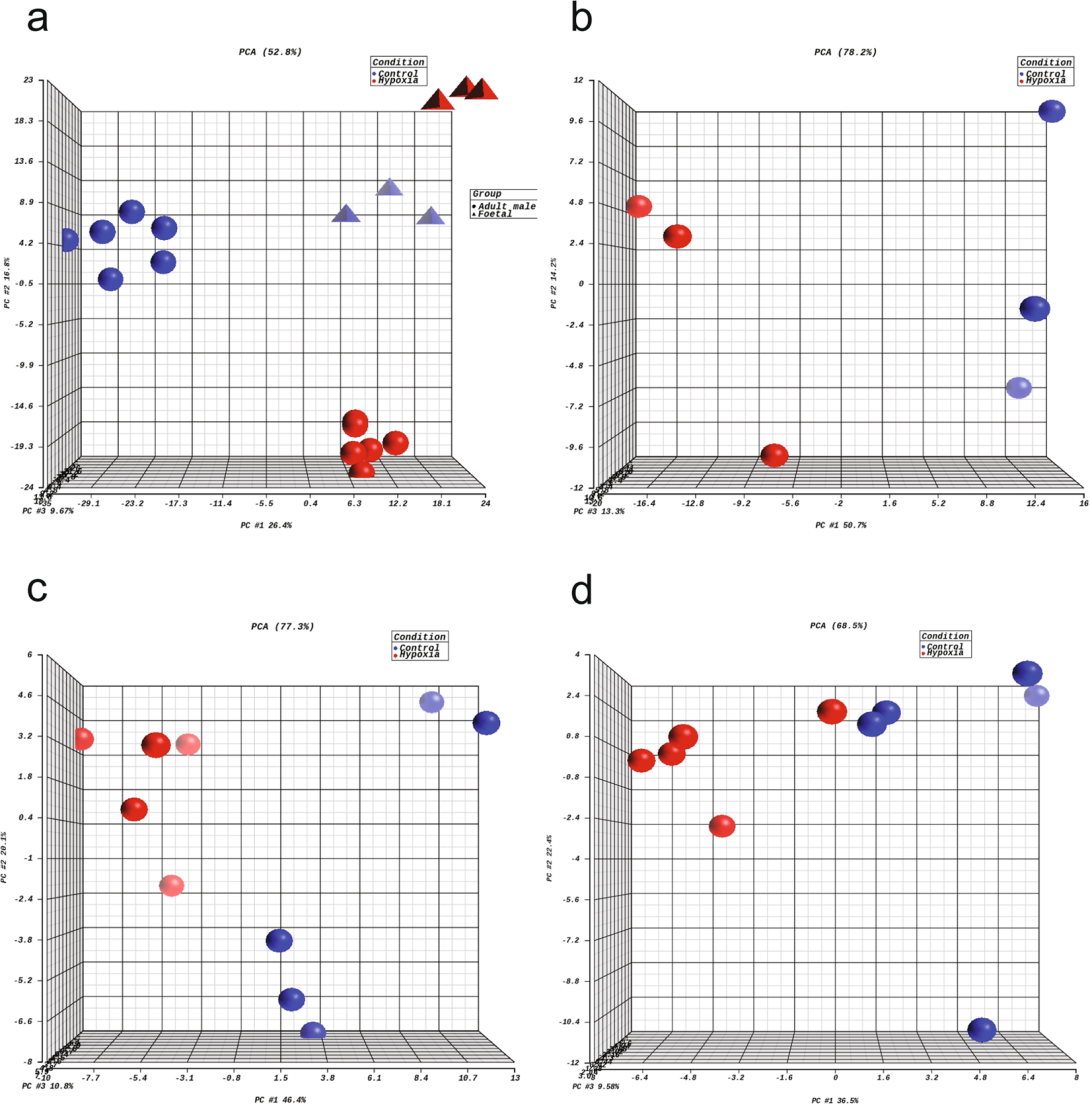
Principle component analysis (PCA) of differential methylation and gene expression profiling. (**a**) Differentially methylated CpGs between control and hypoxia groups in fetal and adult rat hearts. (**b**–**d**) Differentially expressed genes between control and hypoxia groups in fetal, adult male, and adult female rat hearts.

The methylation call files were used as the input for differential methylation analysis using MethylKit^36^ and DMAP^37^. In our original paper, we first used DMAP to process the methylation call files for fetal and adult male rat samples, respectively, to generate CpG region profiles^26^. MethylKit was then used to perform differential methylation CpG (DMC) analysis and differential methylation region (DMR) identification. In DMC analysis, CpGs with a minimum of 10 reads in all samples were considered for the follow-up analysis, whereas in DMR analysis, CpG regions with a minimum of 20 reads in all samples were considered. The criteria to define DMCs or DMRs were based on the false discovery rate (FDR) < 0.05 and the methylation percentage change between control and hypoxia groups were >10%. The principal component analysis (PCA) was performed on DMCs of all fetal and adult male rat samples, showing a separation between control and hypoxia groups in both fetal and adult male rat samples (Fig. 2a).

The gene expression profiles (Fragments Per Kilo base per Million or FPKM) were used as the input for differential expression gene (DEG) analysis using Cuffdiff. Genes with FPKM ≥ 1 in fetal or adult rat samples were used for DEG analysis. In our original paper, the criteria to define DEGs were based on FDR < 0.3 and fold change (FC) > 1.2^26^. The PCA showed a separation between control and hypoxia groups in fetal, adult male, and adult female samples (Fig. 2b–d).

## Data Records

The raw fastq data and processed FPKM gene expression profile of rat RNA-seq data^38^ consist of 6 fetal samples, 10 adult male samples, and 10 adult female samples. The raw fastq data and processed DNA methylation coverage files of rat RRBS data^39^ consists of 6 fetal samples and 12 adult male samples.

## Technical Validation

### Quality metrics for raw and mapped reads of RRBS and RNA-seq data

**Fig. 3:**
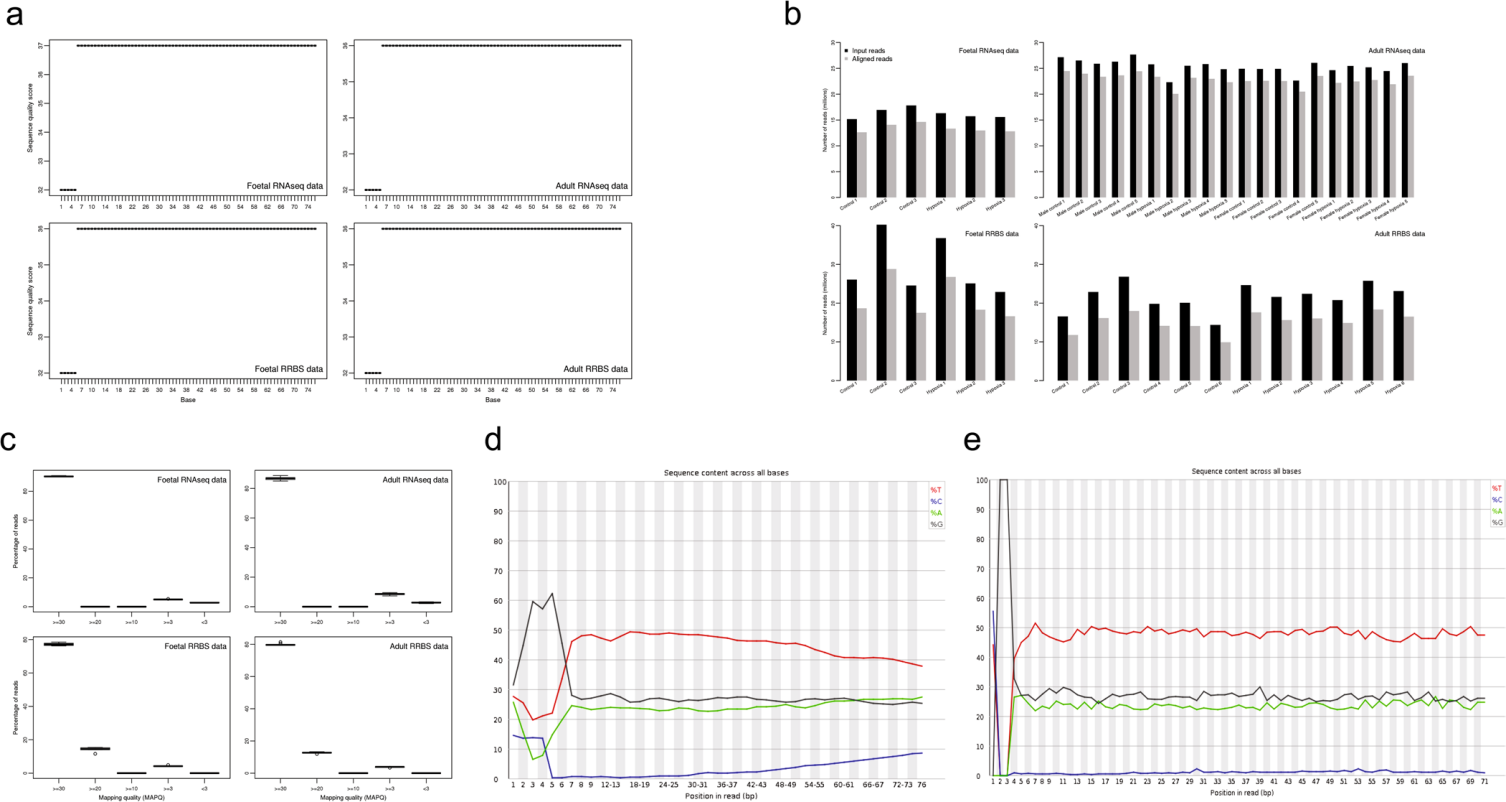
Quality metrics of raw and aligned reads. (**a**) The distribution of median phred quality score per base across all samples in fetal RNA-seq, adult RNA-seq, fetal RRBS, and adult RRBS data sets. (**b**) Number of reads before and after alignment of all samples in four data sets. (**c**) Distribution of percentages of aligned reads across different mapping quality ranges in four data sets. (**d**–**e**) An example of percentage of sequence content per base before and after NuGEN diversity trimming in two RRBS data sets (sample: C1_6).

To measure the quality of raw fastq data, FastQC and ShortRead packages were used to generate QC report for each sample and a general overview for all samples. Figure 3a showed the distribution of median per base quality score for each position across samples in four different data sets, respectively. Usually, the quality scores between 41 and 28 indicate very good quality. In general, the quality scores of RRBS and RNA-seq data in both fetal and adult groups were of reasonable to very good quality. In fact, they showed almost the same patterns across all 76 bases.

Read alignment percentages were summarized in Fig. 3b. Overall, RRBS and RNA-seq data showed very consistent alignment percentages across all samples. On average, the alignment percentages for RRBS and RNA-seq were 64–72% and 89–95%, respectively.

Read mapping quality was measured by analyzing mapping quality scores of the alignments in each sample. Figure 3c presented the distributions of the percentages for aligned reads across different mapping quality ranges. In all of our four data sets, the reads were aligned with high accuracy in all samples, and more than 75% of aligned reads had a MAPQ ≥ 30.

### Validation of NuGEN diversity trimming

Due to the special design of NuGEN Ovation® Ultralow Methyl-Seq Library Systems, the raw fastq data did not show the typical RRBS signature in the first three bases (at the 5′ end) of the reads (CGG or TGG sequences due to MspI digestion, see Fig. 3d). In the trimming process, we disabled option ‘-rrbs’ in trim_galore and further applied NuGEN diversity trimming to remove the diversity adaptors. After the trimming process, we re-ran FastQC, thus the typical RRBS signature in the first three bases can be observed in our QC report for all RRBS samples. (Fig. 3e).

### Performance of ERCC spike-in control

**Fig. 4:**
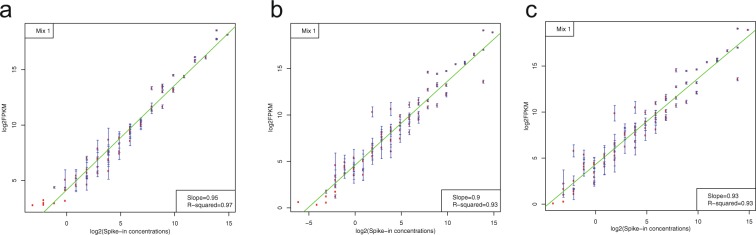
Quality assessment of external RNA spike-in controls (ERCC). (**a**–**c**) Satterplot of linear relationship between log2 transformed true spike-in concentrations and estimated FPKMs (Mean ± s.e.) in fetal, adult male, and adult female RNA-seq data sets.

To access the quality of RNA-seq expression profiles across samples, Mix1 containing 92 synthetic ERCC control sequences was spiked into each RNA sample. Figure 4a–c showed scatterplots of ERCC log2(FPKM) vs. log2(spike-in concentrations) in fetal, adult male and adult female groups, suggesting a good linear relationship between RNA-seq ERCC detected and true concentrations of the ERCC spike-in control. As expected, we observed smaller variability in ERCC with higher concentrations, as compared to ERCC at lower abundances.

### Biological replicates and reproducibility of RRBS and RNA-seq data

**Fig. 5:**
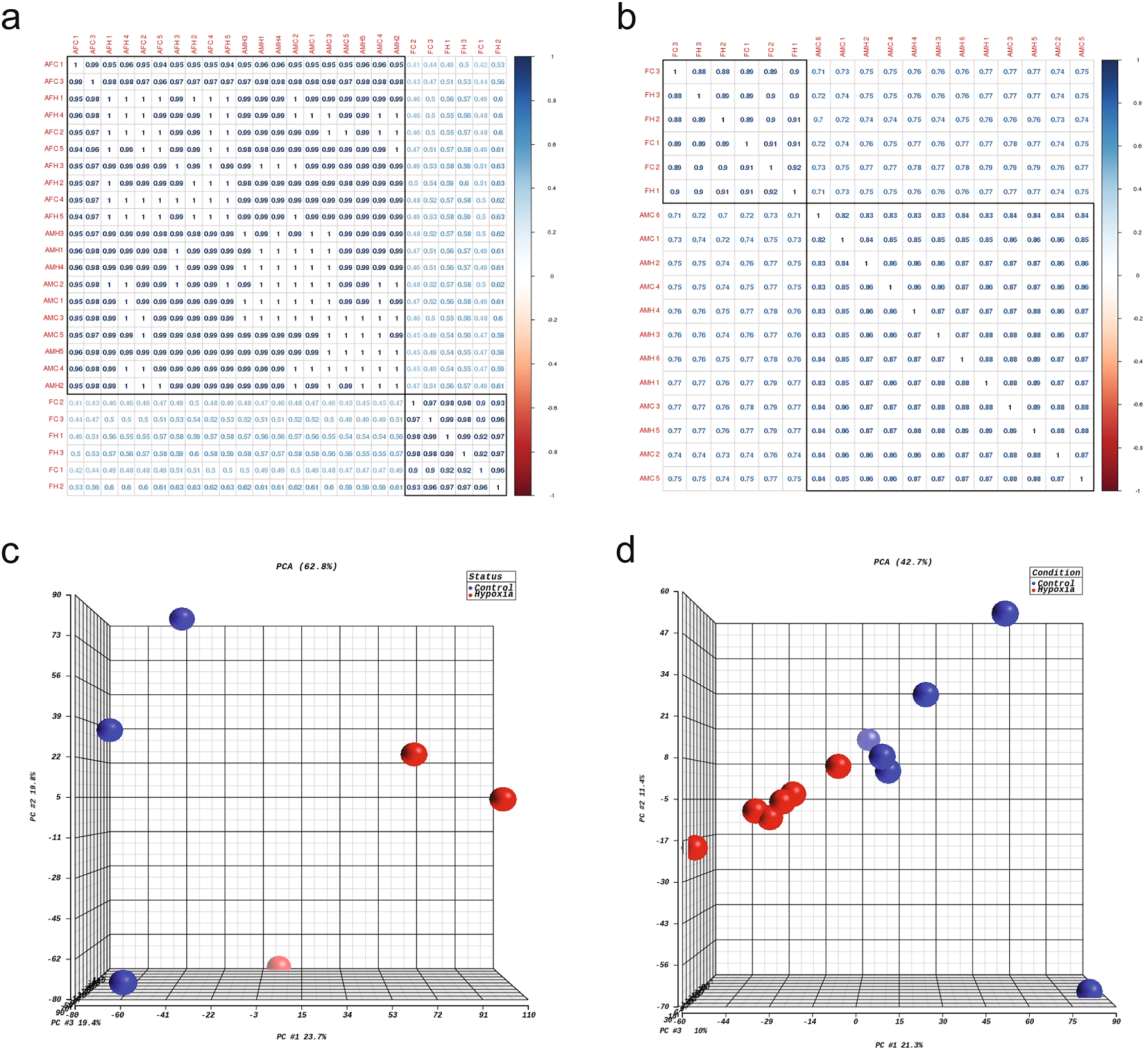
Reproducibility of biological replicates in RRBS and RNA-seq data sets. (**a**,**b**) Pearson correlation and hierarchical clustering of DNA methylation profiles (union CpGs across whole genome) and gene expression profiles. FC, FH, AMC, AMH, AFC, and AFH represent fetal control, fetal hypoxia, adult male control, adult male hypoxia, adult female control and adult female hypoxia. PCA of gene expression profiles in fetal rat heart (**c**,**d**) DNA methylation profiles in promoter region across all genes in adult male rat heart.

Using methylation call files and gene expression profiles, we performed PCA to test the reproducibility of RRBS and RNA-seq data, respectively. The resulting within-group and between-group Pearson correlations were calculated as well (Fig. 5a,b). We observed a clear separation between fetal and adult groups in both PCA and Pearson correlations. Although the clear separation between hypoxia and control groups was not identified in all fetal and adult groups, which may be due to mild hypoxia effect, the biological replicates of fetal RNA-seq data between hypoxia and control groups shared similar variances in PCA (Fig. 5c). Furthermore, we examined the methylation regions located in the gene promoter regions (transcription start site/TSS ± 500 bp) and we found that there were similar variances across samples within either hypoxia or control groups in adult male rat hearts (Fig. 5d).

## Usage Notes

Although DNA methylation plays a critical role in antenatal hypoxia-induced heart developmental programming, much less is known about the global epigenetic variation and molecular mechanisms of this process. Our data sets will allow further analysis to gain a better understanding on the mechanism of hypoxia-induced epigenomic and transcriptomic reprograming and the corresponding impact in the heart development in a sex-dependent manner. Detailed understanding of antenatal hypoxia effect on heart development is of critical importance from both basic and clinical science points of view. The identification of hypoxia-mediated DNA methylation and gene expression biomarkers may provide new insights into potential therapeutic interventions to prevent and treat heart disease associated with fetal stress.

In our original paper, we used classic tuxedo pipeline to process the RNA-seq data^26^. However, the analysis is interchangeable with many other currently available tools. We have re-run the RNA-seq analysis using kallisto^40^ and DESeq2^41^, and we obtained very similar results.

## Supplementary information

Supplementary Table 1.

Supplementary Table 2.

## Data Availability

All tools used in this study were properly cited in the sections above. The settings and parameters were clearly described as well.
