# Supplementary material for: Prenatal hypoxia-induced epigenomic and transcriptomic reprogramming in rat fetal and adult offspring hearts
Source: Sci Data. 2019 Oct 29;6:238. doi: 10.1038/s41597-019-0253-9 (PMC6820751; doi:10.1038/s41597-019-0253-9)
Supplement: Supplementary file 1 — Supplementary Table 1. [file 41597_2019_253_MOESM1_ESM.pdf]

Supplementary Table 1. Statistics Summary of raw, trimmed, aligned reads and CpGs coverage of RRBS data

Sample	Tissue	Raw reads	Reads after trimming	Aligned reads	Aligned perct	CpGs with > 10 coverage	Overlapping CpGs	CpG regions with > 20 coverage	Overlapping CpG regions
C1_6	Heart	26,695,840	26,072,950	18,650,283	69.86%	973,090	590,422	231,354	196,513
C2_11	Heart	40,972,629	40,226,777	28,800,878	70.29%	1,242,160		244,819	
C3_6	Heart	25,062,167	24,535,588	17,498,561	69.82%	972,292		230,393	
H3_1	Heart	37,561,432	36,775,870	26,769,528	71.27%	1,194,791		239,878	
H3_2	Heart	25,559,679	25,089,677	18,294,745	71.58%	1,023,597		232,377	
H5_3	Heart	23,394,623	22,889,181	16,601,333	70.96%	952,553		223,142	
C1	Heart	17,135,032	16,547,962	11,768,295	68.68%	884,322	454,882	203,101	176,402
C2	Heart	24,241,632	22,878,966	16,140,595	66.58%	1,092,544		211,651	
C3	Heart	27,852,951	26,808,469	17,960,090	64.48%	1,126,651		222,460	
C4	Heart	20,749,563	19,790,419	14,118,473	68.04%	1,011,999		212,544	
C5	Heart	20,952,710	20,083,927	14,113,801	67.36%	1,007,384		212,444	
C6	Heart	14,990,291	14,371,737	9,900,370	66.05%	725,525		198,606	
H1	Heart	25,712,725	24,632,094	17,627,567	68.56%	1,119,762		229,018	
H2	Heart	22,462,655	21,585,728	15,623,008	69.55%	1,071,396		217,772	
H3	Heart	23,291,226	22,413,728	16,050,808	68.91%	1,071,479		218,135	
H4	Heart	21,582,884	20,780,342	14,874,035	68.92%	1,028,367		212,191	
H5	Heart	26,982,687	25,777,219	18,327,452	67.92%	1,164,827		222,064	
H6	Heart	23,910,340	23,121,304	16,521,401	69.10%	1,090,146		219,834	
Average		24,950,615	24,132,330	17,202,290	68.77%	1,041,827		221,210	
