# Supplementary material for: Prenatal hypoxia-induced epigenomic and transcriptomic reprogramming in rat fetal and adult offspring hearts
Source: Sci Data. 2019 Oct 29;6:238. doi: 10.1038/s41597-019-0253-9 (PMC6820751; doi:10.1038/s41597-019-0253-9)
Supplement: Supplementary file 2 — Supplementary Table 2. [file 41597_2019_253_MOESM2_ESM.pdf]

Supplementary Table 2. Statistics Summary of raw, trimmed, aligned reads of RNA-Seq data and ERCC spike-in

Sample	Status	Gender	Tissue	Raw reads	Total left reads	Aligned left reads	Total right reads	Aligned right reads	Total aligned pair-reads	ERCC aligned pair-reads	Total aligned reads	Overall alignment rate	ERCC alignment rate	PE
C1-6	Control	Fetal	Heart	34,895,230	15,163,134	13,769,068	15,163,134	13,449,185	12,632,742	24,415	27,218,253	89.75%	0.16%	75X2
C2-11	Control	Fetal	Heart	38,601,636	16,940,751	15,331,154	16,940,751	15,018,240	14,075,483	26,132	30,349,394	89.58%	0.15%	75X2
C3-6	Control	Fetal	Heart	42,102,084	17,800,708	16,072,424	17,800,708	15,613,963	14,642,669	26,028	31,686,387	89.00%	0.15%	75X2
H3-1	Hypoxia	Fetal	Heart	38,231,532	16,311,619	14,617,320	16,311,619	14,286,925	13,323,978	26,657	28,904,245	88.60%	0.16%	75X2
H3-2	Hypoxia	Fetal	Heart	36,522,906	15,711,724	14,257,256	15,711,724	13,804,763	12,971,552	22,875	28,062,019	89.30%	0.15%	75X2
H5-3	Hypoxia	Fetal	Heart	36,385,396	15,575,172	14,041,426	15,575,172	13,675,676	12,801,716	22,879	27,717,102	88.98%	0.15%	75X2
MC3	Control	Male	Heart	58,185,428	27,146,977	26,172,144	27,146,977	25,012,103	24,465,642	80,423	51,184,247	94.27%	0.30%	75X2
MC4	Control	Male	Heart	56,634,526	26,508,245	25,528,656	26,508,245	24,523,356	23,980,726	88,808	50,052,012	94.41%	0.34%	75X2
MC7	Control	Male	Heart	54,916,406	25,886,059	24,877,868	25,886,059	23,949,445	23,386,792	87,580	48,827,313	94.31%	0.34%	75X2
MC8	Control	Male	Heart	56,003,054	26,287,945	25,173,210	26,287,945	24,275,411	23,652,439	81,751	49,448,621	94.05%	0.31%	75X2
MC9	Control	Male	Heart	58,968,558	27,655,885	26,052,099	27,655,885	25,289,436	24,446,787	67,829	51,341,535	92.82%	0.25%	75X2
MH1	Hypoxia	Male	Heart	55,039,114	25,775,880	24,831,468	25,775,880	23,901,996	23,358,079	77,021	48,733,464	94.53%	0.30%	75X2
MH2	Hypoxia	Male	Heart	47,634,206	22,316,457	21,383,592	22,316,457	20,565,900	20,049,445	79,958	41,949,492	93.99%	0.36%	75X2
MH4	Hypoxia	Male	Heart	54,297,082	25,500,117	24,524,929	25,500,117	23,690,788	23,164,253	97,318	48,215,717	94.54%	0.38%	75X2
MH9	Hypoxia	Male	Heart	54,888,454	25,830,422	24,377,736	25,830,422	23,746,863	22,979,753	79,612	48,124,599	93.15%	0.31%	75X2
MH10	Hypoxia	Male	Heart	53,089,986	24,844,176	23,757,890	24,844,176	22,880,421	22,312,129	101,802	46,638,311	93.86%	0.41%	75X2
FC3	Control	Female	Heart	53,455,590	24,919,293	23,962,328	24,919,293	23,013,270	22,544,086	112,624	46,975,598	94.26%	0.45%	75X2
FC4	Control	Female	Heart	53,071,534	24,887,935	23,938,056	24,887,935	23,066,979	22,567,301	119,016	47,005,035	94.43%	0.48%	75X2
FC5	Control	Female	Heart	53,203,616	24,891,294	24,019,361	24,891,294	22,998,780	22,530,308	95,103	47,018,141	94.45%	0.38%	75X2
FC6	Control	Female	Heart	48,201,600	22,630,312	21,745,871	22,630,312	20,955,932	20,484,946	80,985	42,701,803	94.35%	0.36%	75X2
FC7	Control	Female	Heart	55,741,566	26,060,937	25,041,431	26,060,937	24,048,345	23,514,484	102,117	49,089,776	94.18%	0.39%	75X2
FH3	Hypoxia	Female	Heart	52,662,140	24,638,573	23,666,762	24,638,573	22,722,741	22,200,329	99,233	46,389,503	94.14%	0.40%	75X2
FH4	Hypoxia	Female	Heart	54,616,484	25,471,144	23,917,729	25,471,144	22,973,636	22,476,673	130,593	46,891,365	92.05%	0.51%	75X2
FH6	Hypoxia	Female	Heart	53,757,798	25,197,459	24,160,510	25,197,459	23,261,378	22,737,527	97,404	47,421,888	94.10%	0.39%	75X2
FH7	Hypoxia	Female	Heart	52,364,530	24,467,653	23,464,740	24,467,653	22,444,107	21,929,771	81,867	45,908,847	93.82%	0.33%	75X2
FH9	Hypoxia	Female	Heart	55,678,614	26,017,403	25,021,310	26,017,403	24,081,985	23,550,297	100,505	49,103,295	94.37%	0.39%	75X2
